# Delivering public health advice to sign language users: a qualitative study with key stakeholders

**DOI:** 10.1136/bmjopen-2024-098040

**Published:** 2025-09-03

**Authors:** Ruth Rowland, James Bailey, Clare Thomson, Jayne V. Woodside

**Affiliations:** 1 Centre for Medical Education, Queen’s University Belfast, Belfast, UK; 2 Centre for Public Health, Queen’s University Belfast, Belfast, UK; 3 Learning and Teaching Academy, Heriot-Watt University, Edinburgh, UK

**Keywords:** PUBLIC HEALTH, QUALITATIVE RESEARCH, Health Education, Audiology

## Abstract

**Objectives:**

There are more than 10 million deaf or hard of hearing people in the UK. While the deaf and hard of hearing population is heterogeneous, many of those with profound hearing loss are part of deaf communities (UK estimate around 120 000) which are defined minority communities. Many members of deaf communities are sign language users. Studies have shown that health behaviour and knowledge and health-related attitudes and beliefs are suboptimal among deaf and hard of hearing individuals, with reasons not well understood. This qualitative study aimed to explore the effectiveness of delivery of public health messages to sign language users and the potential methods of delivering public health messages beyond direct translation.

**Design:**

Qualitative study, using a phenomenological research approach and using interviews and focus groups. Interviews and focus groups were conducted initially between January and March 2019 and again between September and October 2022. Groups were held where logistically possible. The sessions followed a topic guide developed following review of the literature and discussion with the research team and with patient and public involvement input and pilot testing, but allowed for deviation for discussion depending on the responses given. Interviews took place in either British Sign Language (BSL) or English, depending on the language preference of the participants. Transcripts were analysed using thematic analysis.

**Setting:**

Deaf community and associated stakeholders in Northern Ireland.

**Participants:**

Participants were recruited from members of the deaf community and associated stakeholders across Northern Ireland and sampled purposively to ensure variation in age, sex, language, profession, educational level and region.

**Results:**

There were 16 one-to-one interviews and 5 focus groups held, in total involving 28 participants; 23 females and 5 males. 13 participants used BSL and 15 used English. Ages ranged from 23 to 77 years old. Participants included deaf community members (all BSL users and four English users) and key stakeholders involved in sign language and Healthcare. Interview duration ranged from 21 to 82 min. A number of themes were identified from the transcript analysis. These were broadly categorised into (1) current levels of awareness of public health messages, (2) barriers to accessing public health messages and (3) suggestions for facilitating improvement.

**Conclusions:**

Participants reflected that, as with any heterogeneous population, levels of awareness of public health messages vary widely across Deaf communities. Overall levels of awareness were felt to be generally low and certainly much lower when compared with the hearing population. Particular difficulties were noted with regard to mental health, more abstract health-related concepts and preventative health measures. Participants identified not only communication barriers but also systemic, cultural and attitudinal barriers as contributing to this. Suggested next steps involve implementing legislative reforms to address systemic barriers, conducting awareness training to tackle attitudinal barriers, and launching culturally appropriate public health campaigns, all of which should be deaf-led to ensure the expertise and lived experiences of Deaf people guide the process.

**STRENGTHS AND LIMITATIONS OF THIS STUDY**

- The study used best practice in terms of provision of information about the study and gaining of informed consent (with all materials available in British Sign Language (BSL) as well as English).
- Researchers had relevant research experience and were also qualified BSL interpreters, which allowed for direct communication during interviews without the necessity for interpreters.
- Purposeful sampling achieved a diverse cohort of participants with wide variation in age, language, profession, educational level and region.
- Interviews took place in BSL which the research team chose to translate to English for subsequent transcription and write-up. Therefore, analysis was conducted in a language in which not all interviews took place, which poses inherent challenges (eg, linguistic understanding and cultural representation).
- All fieldwork was conducted in Northern Ireland, where there is the added complexity of sign language users employing either British or Irish Sign Language as their first language; given resource constraints, stakeholders were included from the predominant language used, that is, BSL, but it has to be considered that views may differ among these two different communities.

## Introduction

 Deaf and hard of hearing people make up a substantial proportion of the population. There are more than 18 million deaf or hard of hearing people in the UK, with 1.2 million of these having profound hearing loss.^1 2^ While the deaf and hard of hearing population is heterogeneous, many of those with profound hearing loss are part of deaf communities which are defined minority communities.^3^ As with any cultural and linguistic minority group, deaf communities have their own unique rich culture, artistic expression and heritage. The commonality of shared experience is a strong unifying factor for the community and members identify a deep sense of identity and belonging. Many members of deaf communities are sign language users, with both British Sign Language (BSL) and Irish Sign Language (ISL) being prevalent in Northern Ireland. Figures estimate that approximately 18 000 BSL and ISL users live in Northern Ireland, 7500 of whom are Deaf.^4^ Both BSL and ISL are recognised languages with their own grammatical structure, syntax and lexical items and are not dependent on or strongly related to spoken English.

Many users of signed language experience difficulties in accessing healthcare^5^,^8^ and have a poor understanding of healthcare, including public health recommendations around disease prevention and healthy lifestyle advice.^9^ Studies have found deaf people’s health to be poorer than the general population, with probable underdiagnosis and undertreatment of chronic conditions putting them at risk of preventable ill health.^9^,^11^ Studies of health behaviour and knowledge and health-related attitudes and beliefs show this to be suboptimal among deaf and hard of hearing individuals.^910 12^,^16^ The reasons for this are not fully understood, but certainly opportunities to access information about illness prevention, diagnosis and treatment are more limited. Language deprivation and audism are likely to be causal mechanisms of poorer health literacy and the presence of health inequities among deaf people. Many deaf individuals grow up without the ability to overhear spoken conversations, environmental sounds or other auditory cues that contribute to everyday knowledge acquisition. This results in a reduction in incidental learning, which refers to informal, unplanned learning that occurs through exposure to information in one’s surroundings. This depletes what has been referred to as the ‘fund of information’, a concept which describes this accumulation of general knowledge over time.^11^ Low levels of health literacy have been shown to directly impact on the health outcomes of users of signed language.^11^ Sign language users also face difficulties accessing healthcare information that is usually presented in English, similar to members of other communities who use English as a second language.^17^,^19^ They have an additional barrier of often not being able to communicate directly with their healthcare professional, and poor cultural competency and awareness are being reported to frequently result in misdiagnosis and referral.^10 13 20^ Communication barriers are likely to be a principal cause, but cultural and other factors may also contribute.^9 11^

Suggestions to improve the delivery of public health and other health information have been made,^11 21^ largely focused on direct translation into signed languages using videos,^22 23^ but also making text simpler,^24^ e-communication and online education programmes^21 25^ and exploring health information accessibility.^26 27^ However, few studies have formally tested the effectiveness of such an approach on health behaviours and outcomes.^11 21 28 29^ A recent systematic review, focusing on health outcomes more broadly, but which offers valuable insights, highlights that there has been a focus to date on the translation, validation and identification of clinical tools and scales,^30^ which is an important early step in conducting such studies of effectiveness.^11^ The same systematic review highlights a focus on healthcare provider training, but also enforces that meaningful engagement with deaf individuals is imperative during the development and evaluation of such interventions, in order to achieve more equitable care for deaf communities.^11^ Few studies until recently^11 31 32^ have engaged directly with deaf communities to determine what approaches are preferred and are therefore likely to be the most effective in terms of knowledge and behaviour change, and whether cultural adaptation of the messages would enhance their effectiveness.^33^

This qualitative study, using a phenomenological research approach, aimed to explore how to most effectively deliver public health messages to sign language users and the potential methods of delivering public health messages beyond direct translation. This question was discussed with key stakeholders in deaf communities, involved in sign language or healthcare using small group discussions and interviews. The work was conducted in Northern Ireland.

## Methods

Given the ambition of developing practical understandings of concrete, real-world issues around public health messaging in deaf communities, the philosophical orientation of this research approach was pragmatism, with a range of stakeholders, both from deaf and hearing communities included.

Participants recruited were members of deaf communities and associated stakeholders across Northern Ireland. Participants were sampled purposively to ensure variation in age, sex, language, profession, educational level and region. This included members of deaf communities, sign language translators and interpreters, children of deaf adults, social workers working with deaf communities and representatives from the British Deaf Association, Royal National Institute for Deaf People, National Deaf Children’s Society, the Mental Health and Deafness team, and the Public Health Agency. Snowball sampling was also used to identify further participants. Recruitment was conducted by researchers embedded in and known by deaf communities. The sample size was not predetermined. The aim was to maximise a range of views and to conduct interviews until data saturation was reached, that is, when the same themes or patterns were emerging repeatedly during interviews/focus groups, with consideration given also to ‘information power’, that is, ensuring that the data collected provided sufficient depth and richness to address the research question, although neither of these was formally evaluated. Initial recruitment was via direct contact with potential participants, sending an initial invitation letter, followed by an information sheet being provided to those who expressed interest and which explained the reasons for doing the research and what would be required. This was available in both English and BSL. Written informed consent was obtained from each participant before interview. Participants were reminded that participation was voluntary and they could withdraw at any time, without explanation.

Interviews were conducted initially between January and March 2019 (conducted by JB; male) and again between September and October 2022 (conducted by RR; female). Both individual semistructured interviews and small group discussions were used depending on logistics or stakeholder preferences. Sessions were conducted face to face or online, again depending on participant preferences and logistics. No-one else was present during data collection other than the individual researcher and participants. The sessions followed a topic guide developed following review of the literature and discussion with the research team, but allowed for deviation for discussion depending on the responses given. The topic guide was pilot tested by the research team (duration of session; whether questions were understood) before being used for data collection. The topic guide was updated prior to the second set of interviews, to include questions relevant to the COVID-19 pandemic and associated change to service provision, in the interim period. Data collection and analysis took place concurrently to allow for findings in the early discussion groups to inform areas explored in the later ones. Initial interviews were conducted by one qualitative researcher (JB) and a second set of interviews conducted post-COVID-19 pandemic was conducted by a second qualitative researcher (RR). No repeat interviews were conducted. Both researchers were employed by Queen's University Belfast (QUB) at the time of data collection and had previous experience of conducting interviews and focus groups, but also received refresher training within the research team. Interviews took place in either BSL or English, depending on the language preference of the participants, but each individual session took place in only one language (there was no interpretation within the session). Interviews were audio-recorded or visually recorded depending on the language being used and transcribed verbatim. BSL/English interpreters were used to translate the interviews conducted in BSL, for transcription purposes. Transcripts were not returned to participants for comment and/or correction; this was done by researchers only. Researchers also made field notes. All data were collected and stored in line with current General Data Protection Regulations.

Transcripts were reviewed line by line by two researchers (RR and JB) to ensure deep familiarisation with the scope and nuances of the qualitative data. Following Braun and Clarke’s six-phase approach to thematic analysis,^34^ the researchers conducted inductive coding, identifying recurring words, phrases and concepts that reflected participants’ lived experiences. Initial codes were compared with identifying patterns and constructing preliminary themes. These themes were then discussed with the wider research team to assess their coherence, relevance and where there was sufficient supporting data for each theme. Through iterative discussions—including input from JW—discrepancies were resolved, and themes were refined to ensure they authentically represented the data. The final overarching themes were confirmed once consensus was reached on their interpretive validity and alignment with the phenomenological focus of the study. Data were managed in Excel, as the scale of the study made this manageable and not all team members had access to NVIVO smaller-scale studies or when the qualitative data were relatively straightforward. Participants did not provide feedback on findings prior to submission for publication; dissemination is planned in future.

### Patient and public involvement

Public involvement (members of deaf communities and other stakeholders involved in public health messaging in connection with deaf communities) was involved in the conduct of this research in that the research question was developed in consultation with deaf communities (through one-to-one contact and discussion), research team members directly conducting data collection were drawn from those eligible to participate, and the developed topic guide was pilot tested with those eligible to participate and feedback incorporated.

## Results

There were 16 one-to-one interviews and 5 focus groups held, involving 28 participants: 23 females and 5 males. 13 participants used BSL. 15 participants used English. Refusal to participate was limited (<10%) and related to time concerns or simply not wanting to engage with the research process. Ages ranged from 23 to 77 years old. Participants included deaf community members (all those who used BSL during data collection and a further four, being children of deaf adults, who used English) and key stakeholders involved in signed languages and healthcare. Interview duration ranged from 21 to 82 min. No participants withdrew their consent. Researchers agreed data saturation was reached and information power was sufficient.

A number of themes were constructed from the transcript analysis. Some of these are related to healthcare access more generally, early life experiences, language acquisition, lack of incidental learning and the impact of the COVID-19 pandemic. While these themes are not directly related to public health message delivery, it is important to note that participants commonly recognised early life experiences as contributing to difficulties accessing public health messages both at an early age and with subsequent impact in later life, and it was frequently observed that these early difficulties served to perpetuate all other contributing factors. The themes were broadly categorised into (1) current levels of awareness of public health messages, (2) barriers to accessing public health messages and (3) suggestions for facilitating improvement.

### Current levels of awareness of public health messages

#### Current low levels of awareness of public health messages among users of sign languages

It was generally felt that current levels of awareness of public health messages around sign language users were low, and certainly much lower than that of the hearing population. Inequality of access to and awareness of public health messages was a common area of discussion, and participants readily compared their experience with that of hearing individuals. Recollection of the COVID-19 pandemic highlighted the perceived discrepancy of experience.

I suppose during the day hearing people could have watched live videos, learnt about the death toll and the daily stats, but Deaf people had to wait until the information went out in the evenings, so we had to wait a bit longer. (Deaf community member)

The lack of support for hearing people is bad but then for Deaf people we are scraping the barrel. (Stakeholder)

While generally, health awareness was felt to be low, a number of areas were identified as being particularly challenging. These included mental health awareness, awareness of more abstract health-related concepts and preventative health awareness. A number of participants noted a tendency to only seek out health-related information, which was felt to be directly related to the individual, for example with regard to a condition they were diagnosed with. Participants hypothesised that this related to the intentionality with which sign language users are required to access health messages, which will later be discussed. Unfortunately, such health behaviours can lead to poor awareness re: health prevention and delayed diagnosis.

I think some Deaf people may not act upon certain symptoms that may come up, either through embarrassment or lack of knowledge and maybe don't go for the early intervention. By the time they do go… it’s much further down the line. (Stakeholder)

If you don’t have that early intervention at health promotion, then things can go horribly wrong later on, and at a very much worse stage. (Stakeholder)

Participants shared a number of anecdotal examples, reflecting gaps in health awareness, with potential for serious impact on health outcomes.

I take the blue one at dinner and that’s insulin. What’s the grey one? I couldn’t tell you to this day. As far as I’m aware it’s a stabiliser. Maybe not. The other one is insulin. Still to this day I don’t know. (Deaf community member)

I knew and she kept telling me she was a gluten-free diet. And I kept seeing her eating bread. And about two years later, she came and said I’ve just discovered that wholemeal bread isn’t gluten-free. (Stakeholder)

I told her she was drinking too much Coke and the caffeine levels were dangerous for the baby but she was totally unaware of this as the information hadn’t been made available to her. (Deaf community member)

A striking and significant finding is that many health-related terminologies were noted not to currently exist in BSL, which may reflect the limited awareness or discourse surrounding the concepts within deaf communities.

There’s a lot of difficulty understanding the language around these conditions especially as these lexical items don’t exist yet in Sign Language. (Deaf community member)

#### Sign language users are not a homogeneous group and so awareness varies

Participants reflected on the variation in current levels of awareness of public health messages among sign language users, acknowledging that sign language users are not a homogenous group, and so, as with any population, levels of awareness of public health messages vary widely across the community. A number of factors were suggested to contribute to such variation, including: differences in educational background and early language acquisition, ability to access English as a second language, family structure, age, geographical region, employment and generational differences.

 like in the hearing community, it (public health awareness) varies enormously. (stakeholder)

I think some of it will be better life experiences, what people are exposed to early on and their early context of growing up Deaf. I suppose, and what’s being provided around them. The scaffolding that that’s provided around them… it’s dependent very much on the context that you’re in. (Stakeholder)

In particular, participants reflected on generational differences in awareness of public health messages. This was attributed in part to historical and cultural factors surrounding lowered provision of and therefore expectations of access to information, but lack of ability to access advances in more accessible technology and social media campaigns was felt to be the biggest contributing factor to this variation.

I don’t like to kind of put a label on one section but I do think that older people find it more difficult to access information than younger people because they’re used to having the information given to them… in certain formats and it hasn’t really worked. (Stakeholder)

For younger people it’s social media and each other—they find technology more accessible, and they can, they seem to be more aware. For the middle aged and older group, using social media is much—they may be able to use mobile phones to text each other, they may be Skyping each other, but in terms of getting public health information I don’t think they’re actually going on to sites to use it for that purpose. (Stakeholder)

Unfortunately, participants who reflected on the variation of levels of public health awareness within the population indicated that it is often those with the lowest levels of health awareness who are subsequently further excluded from accessing information.

It’s those people who are left behind. If you’re already behind, you are going to be even more left behind. (Stakeholder)

#### Sign language users access public health messages in a range of ways, but accessibility is low

Participants indicated low expectations surrounding ability to access public health messages as contributing to reduction in health-seeking behaviours around public health messages. Repeated exposure to structural barriers to accessing information was felt to impact directly on subsequent health behaviours and attitudes, creating a culture which does not actively seek out health information. It was felt that an attitudinal and cultural shift would therefore be required in addition to systemic changes in order to improve awareness of public health messages.

Sign Language users traditionally are not engaging with those services and are not actively seeking out information because they just assume it’s not accessible to them and in many cases they are right and so they don’t even try to find out. (Stakeholder)

they often meet a barrier, so there is a certain reticence and an acceptance on behalf of Deaf people, well it’s not for us anyway and never has been. (Stakeholder)

information out there that’s accessible doesn’t really matter if you don’t know to go and look for it somewhere, if you don’t know that such a thing exists as getting access to information. (Stakeholder)

Information sessions, organised by deaf organisations or health services, were commented on as one of the most prominent and effective current means of sign language users receiving public health messages. The ability to take sessions at a controlled and participant-directed pace, perhaps led by a deaf facilitator and with subsequent opportunity for discussion, was noted to be particularly beneficial. Indeed, anecdotes were shared of subsequent diagnosis or health behaviour change following a session, highlighting their benefit. However, participants also noted limitations of such sessions, which included the perception that often participants were from a similar demographic and sessions did not access harder to reach members of the community.

These provide the space for Deaf people to learn in their own language, ask questions and have discussions. (Deaf community member)

And, once we had started the programme, it was very concerning that we immediately followed up the first night’s…with two visits to the GP the next morning. (Deaf community member)

There are more opportunities for Deaf people to attend information sessions… which is excellent but the Deaf people who attend these are the assertive, well-connected members of the community. The wider, harder to reach community aren’t being reached. (Deaf community member)

Word-of-mouth was discussed as a frequent means in which public health messages are relayed among sign language users. Participants gave examples of this including: informal conversations between friends, discussions at deaf community groups and deaf people sending video messages to up-date others. Participants reflected on both the positive and negative aspects of this. On one hand, it was felt that conversations among sign language users were an effective way to ensure messages were communicated in a culturally appropriate and comprehended manner. Participants felt this type of communication often was the most penetrative, often resulting in behaviour change with sign language users more likely to implement the message and repeat it to others. Social media was largely recognised as an increasing way in which such personal messages are being disseminated and was felt to be particularly impactful, especially for younger generations. However, participants also recognised the potential pitfalls of this, in terms of potential for spread of misinformation, especially within a small community. This is of particular concern in a community that does not have equitable access to other sources of information, which are necessary to filter new information and make a judgement on its reliability. Thus, misinformation can be quickly amplified among the community.

The most effective way of learning about conditions etc is from other Deaf people but then you run the risk of the information not being completely reliable or correct. (Deaf community member)

because there’s so much misinformation or misdirection, again it is siphoning through that without that ambient information that you've grown up with. (stakeholder)

Participants highlighted that often users of signed languages access public health messages directly via the professional they are interacting with. This can result in large variation and unreliable, inconsistent access to public health messages. This was felt to be dependent both on the healthcare professional and the interpreter. Participants suggested that professionals lack cultural understanding and sensitivity and are not aware of accessible resources, therefore, unable to effectively sign-post sign language users. A lack of shared understanding of concepts and life experiences was noted to be a particular barrier in terms of effective communication between a hearing professional and deaf sign language user.

The life experience; the parameters that are drawn upon to get to what is seen as the norm from the hearing world and the deaf word. You actually need to also remember that, that this person’s life parameters for their understanding are slightly different. (Stakeholder)

I think most people I come across only get it from the doctor that’s in front of them. So, they get as much time as they get and that depends on the communication skills of the doctor, and it depends on the communication skills on the interpreter in front of them too. (Stakeholder)

that’s why these health messages need to come, not just through the face-to-face stuff. I think it’s really crucial because not all interpreters are consistent. (Stakeholder)

### Barriers to accessing public health messages

#### Sign language users experience a lack of incidental learning opportunities which impact on overall awareness of public health messages

Participants contrasted the experience of deaf individuals with hearing individuals who have access to unintentional incidental learning, throughout life. This affords hearing individuals the opportunity to subliminally acquire and consolidate on public health messaging, frequently and gradually. Lack of access to radio messages, corridor conversation and television adverts or storylines reduces the opportunity for a deaf sign language user to do this in a similar way. Lack of incidental learning was noted to hinder development of generalised schema surrounding health, making it difficult to acquire and contextualise new information readily.

 When you’re Deaf you miss out on so much that hearing people take for granted: chit chat, radio programmes, newspapers and as a result there’s a lot of information that just isn’t available if you’re Deaf. (Deaf community member)

The hearing people would think oh yeah you know this because of this and it’s linked to this. They already have this back log of knowledge. But for Deaf people it’s passed on information with no background. (Stakeholder)

In contrast to the ease of incidental learning afforded to hearing individuals, participants frequently highlighted the intentional nature required in order for a sign language user to seek out health information; whether that be attending an information session or purposefully searching for accessible content online.

A hearing person, wouldn’t get in (the) car and drive all the way to Belfast. I’d listen to it on the radio or a podcast. We’re all busy and have lives. It’s a big commitment to go and sit to hear about something that a hearing person has had access to, all their lives. (Stakeholder)

any information to do with health issues, the Deaf community are expected to go to a planned event but deaf people want to be able to sit in their own house and access information like hearing people can. (Deaf community member)

Therefore, sign language users often access public health messages on a one-off or ad hoc basis. Participants highlighted that this cannot be expected to equate to the gradual build-up of knowledge acquired via repeated exposure.

I think the general public is getting this message repeatedly and Deaf people aren’t, so I don’t think a one-off video would help. (Stakeholder)

 it just makes it a bit more consolidated when they’ve heard it more often. (Stakeholder)

#### English as a second language reduces the impact of written literature which is often relied on in public health

Public health messages often rely on written literature, for example, in the form of leaflets or posters. Although this will not be the experience of all sign language users, participants frequently reflected on both the significance of this barrier and professionals’ lack of awareness regarding this.

It all comes down to the fact that any published information that is distributed just isn’t accessible. Accessible for Deaf people means they are able to watch and digest the information it in their own language however as it stands the norm is that all information is in written English, thus Deaf people are unable to understand or access it completely. (Deaf community member)

I think it would be fair to say that the root of it is that a lot of the other professionals out there don’t realise that a Sign Language user doesn’t necessarily understand written English…therefore (think) it’s okay to give them written information. (Stakeholder)

Even those participants who felt able to confidently use English as a second language expressed preference for first language access.

I have a good level of English but good English isn’t enough. It’s also about not feeling 100% confident in my understanding as I’m relying on my second language and as it’s about my health it’s not an area I’m willing to take any chances on. (Deaf community member)

trying to understand something in your second language is hard work and off putting. (Deaf community member)

While ensuring English information is translated into sign language may appear a suitable solution to the above, participants were clear that, while this is necessary as a minimum, it is not adequate. Many participants referred to the limitations of this approach, including sign language users not knowing where to access resources which have been translated, translations being deemed uninteresting and, vitally, a direct translation being vastly unable to account for the potential existing gaps in a sign language users’ schema surrounding public health messaging, given the issues previously discussed.

BSL translations, it’s great to have access, but I wonder how many Deaf people are actually accessing them…I don’t think that would be anybody’s first point of call- oh I need to know this information, I’ll look for a translation on a website. (Stakeholder)

It’s impossible for an interpreter to be able to fill in absolutely everything that’s lacking in the incidental knowledge gaps of Deaf people. (Deaf community member)

If an English text is merely translated it still won’t be comprehensible or ideal. (Deaf community member)

#### Barriers in health service provision exist which are public health messages-specific

While general access to health service provision was noted as an issue, barriers to service provision were also noted to prevent sign language users from following up on public health messages which they have been able to access. Participants reflected on health messaging campaigns often advising follow-up action such as phoning a support line or seeking additional support. Given that these avenues are frequently inaccessible, it was felt that this portrayed the message that sign language users are excluded from such public health messages and so contributed to disengagement with such concepts entirely.

Oh, yeah, I could see the helpline at the end of the episode whatever call the helpline, but that’s all no use to me, I couldn't do that because I can’t access that. (Stakeholder)

Let’s say there was one on alcohol misuse. You could look through that and go, yes, that’s signing, I’m drinking too much here, I need help with this. You then go to the health service…but then you’re told, we are charitable organization and we can’t afford an interpreter, and therefore it stops there. (Stakeholder)

The likes of the UTV campaign about looking after your health has made Deaf people think twice but even if Deaf people look for support it’s very hard to find any that’s accessible. (Deaf community member) [AUTHOR NOTE: UTV is a local television channel]

### Suggestions for facilitating improvement

#### Legislation required to underpin any attempt at improving public health message delivery and accessibility

Participants acknowledged the requirement of legislation to fundamentally underpin any attempt at improving delivery of public health messages. It was frequently acknowledged that subsequent attempts at improving delivery of the message itself would not be successful without first addressing fundamental issues with language acquisition, education, signed languages legislation, ensuring continued funding for deaf organisations and focussing on systemic issues such as barriers to accessing healthcare provision itself. Those interviewed highlighted hopes that systemic and cultural change would also lead to increased training and awareness for healthcare professionals.

I think the first step would perhaps be to make Deaf awareness training mandatory for every single member of staff who works in the hospital. (Stakeholder)

As much as there is online training, it’s not mandatory, and I do think that that could be improved. We have such a lot of mandatory training requirements, and the information is there, it’s being drawn up, it’s online, but there’s no obligation of all staff to complete. So, down the line, it would be good to see that. (Stakeholder)

We need more Doctors willing to go and learn Sign Language. That will improve Deaf people’s access to services and general health knowledge. (Deaf community member)

#### Accessibility to public health messages required only as a minimum

Discussions surrounding improving access to public health messages often focus on accessibility of the message. Participants highlighted that this is necessary but as a minimum, and accessibility alone should not be considered adequate in terms of effective delivery of public health messages, with emphasis that access does not necessarily equate to understanding.

There have been some improvements and more resources are being translated into Sign-Language but we’re still not there yet. It’s not just a case of having information interpreted, Deaf people need more than that. (Deaf community member)

However, participants did emphasise the need for access as a minimum and as part of a broader approach. They highlighted potential benefits of QR codes being attached to leaflets and posters, allowing access to a video translation, and emphasised the importance of this translation being available in both BSL and ISL.

Ideally there would be a QR code that would bring you to a signed format of the leaflet. (Deaf community member)

if we’re going to get it right, you may as well get it really right and have it in ISL as well as BSL. (Stakeholder)

Participants suggested the establishment of a single point, which collated accessible resources, that sign language users could be directed to, reflecting that currently there is unreliable haphazard utilisation of translated resources.

It would also be good to have details of conditions, self-management tools, health professionals all in one place. (Deaf community member)

it’s great to have access but I wonder how many Deaf people are actually accessing them. Unless it comes up on their newsfeed, on Facebook, on something they are specifically looking at, then it just gets lost and then what (Stakeholder)

Participants believed all public health messages broadcast on television should be accompanied by signed languages translation, alongside improved subtitling. This was felt to not only have benefits in affording access to direct public health messages via Public Health Agency adverts, but also the more indirect messages that come with viewing a health message in a TV programme or the health information that can be gained from watching a documentary. Some participants felt this approach may be particularly useful for an older generation, who may watch television more than using online resources, and provide a level of continuous incidental messaging, which sign language users often miss out on.

I think all public health adverts should have an interpreter, a Deaf translator on them, absolutely…

the older generations are watching TV and they’ll see that and have access to that information every day. (Deaf community member)

…because most information about health actually comes peripherally, it comes from conversations, it comes from … they do programs. It comes from watching the TV program and engaging with characters going through a health journey. (Stakeholder)

#### Specifically created and targeted campaigns for users of signed language

Throughout the interviews, participants recognised the need for public health messages to be specifically created and targeted directly with sign language users in mind. Many participants felt that current messaging was simply translated as a ‘second thought’ and equated this to tokenism. They reflected on the contrast between this and campaigns aimed at a hearing audience being well planned and thought out, in order to best address the target audience.

there’s a lot of thought goes into marketing the general public health messages, so ‘FAST’, you just know about it. They think about how to make it easy for the general public to access. It has to be quick and easy to remember. They come up with slogans and fancy stuff. There’s so much thought that goes into public health messages, but for Deaf people it’s almost as a secondary thing they’ll just translate it, give no thought to the audience. But for the general public they give so much thought. (Stakeholder)

This is tokenism at its finest! This is a mainstream piece being translated to Deaf people and it’s not at all engaging. It needs to be written for and presented to Deaf people. (Deaf community member)

Similarly, participants noted the need to target specific groups of sign language users with specific campaign strategies, in a similar approach that is taken for the hearing audience, as opposed to grouping ‘users of signed language’ together as one supposedly homogenous target audience.

It’s not just ‘the Deaf community’. It’s people who have different levels of understanding, it’s different ages. So, it reflects the hearing community in that way. It’s not, just because you’re Deaf, it’s not one size fits all. (Stakeholder)

There’s a broad wealth and diversity of what is right, or what’s appropriate within a group of people that are not homogenous; they’re heterogenous. (Stakeholder)

That is the thing. So, what’s appropriate for Deaf people, it will be appropriate or relevant for some, but not for all. I think one of the things about hearing populations that messages are given a different way to accommodate the range of the abilities… because if you just produce one size fits all, it won’t work. (Stakeholder)

The importance of making specifically targeted campaigns culturally appropriate was emphasised repeatedly throughout the interviews. Utilisation of visual imagery and concepts familiar to grammatical features of signed languages was suggested as ways to achieve this. Almost all of the examples of public health messages, which participants cited as being impactful, featured strong visual imagery.

It needs to be culturally appropriate and paced correctly so that it is engaging and effective for Deaf people. (Deaf community member)

Well, I think the ‘Take Five.’ I don’t actually know if it was a public health message… but, it was condensed into five bullet points and it was about preserving your own mental health… there’s five things that you can do that will aim to improve your mental health…That worked well for everybody. But, in particular, for Sign Language users because you can be quite visual… five, you can gesturize [sic] five, and then one, two, three. That’s very, very close. Categorizing things into numbers is very, very close to how Sign Language users like information to be conveyed. (Stakeholder)

it also needs to be visually excellent. It can’t have jargon or be too text heavy, instead it needs to be visual. Again, that FAST advert is a format that I would suggest and it’s beneficial to everybody because it’s very simple, very visual and the impact is great as it’s very memorable. (Deaf community member)

#### Deaf people need to be at the forefront of the development of public health message development

In order to achieve any effective intervention, participants emphasised the necessity of deaf sign language users taking the lead on design and consultation, and being at the forefront of any proposed change.

Ideally the information would be …in a format that has been decided and led by a Deaf person. (Deaf community member)

I think most of those messages are lost because they’re not designed with any input from the Deaf community. (Stakeholder)

It was highlighted repeatedly that, for a multitude of reasons, sign language users relate to sign language users and so will naturally respond better to messages, which are designed by and feature them.

Deaf people are the experts in their own lives so it needs to be a Deaf person in that role. And Deaf people resonate with another Deaf person because ‘you’re the same as me’, its an instant thing, it’s a culture, it’s a community. (Stakeholder)

Suggestions for content featuring sign language users ranged from utilisation of deaf characters in television plotlines or creating advertisements or social media campaigns featuring role-plays with deaf characters to campaigns including use of personal stories coming directly from deaf community members. Throughout interviews, participants strongly favoured information directly conveyed in signed languages, rather than a translation, with particular preference for use of personal anecdotes or experiences.

I would like a Deaf person to make a video about their own personal experience. So for example, if someone been through cancer, something like that, I think…that would be an easier way to take in that information in a direct and understandable way. (Deaf community member)

If shows like that were more proactive in bringing in Deaf characters and allowing them to actually go through a storyline in a productive way, rather than as a novelty (Stakeholder)

I think, people would buy into it a lot more quickly. It’ll bring that sense of, yes, I’m entitled to this information. (Stakeholder)

I think having a mock video with actors in it, showing exactly the process of a health scare or whatever it may be, I think that’s a really good way to help us understand it. If we do it with an interpreter or captions, that’s all good and well… but I think having it actually played out is a really good way of conveying the message. (Deaf community member)

Participants reflected on the potential benefits of increasing access to and encouraging training of deaf professionals within health infrastructure itself and suggested the potential development of a liaison role, whereby a deaf person is able to liaise with public health agencies and advise on public health messaging. In addition to this, multiple participants noted the benefits of both deaf interpreters/translators and deaf relay interpreters and encouraged ongoing development of these roles. Deaf relay/intralingual interpreters (sometimes referred to as deaf relays) are qualified deaf interpreters who work alongside hearing interpreters with users who are deaf and have a specific language need. This could be due to a disability, mental health condition, limited language development or being a non-native language user. Benefits such as the ability to clarify concepts or decode complex language in a culturally appropriate manner were highlighted. In order to see further development of these roles, increased training provision will be required.

putting Deaf people at the front of health promotion pretty much and not only within the informal through soaps and things like that but within the professional world. (Stakeholder)

I don’t think there are enough Deaf professionals promoting the health message in whatever area or sphere and maybe that is an area that needs to be addressed through education through the programs, whether it’s nurse programs, psychology programs, medical programs, to encourage Deaf people into those areas…you’re getting people who are then seen as role models and health promoters just by the fact that they’re in a health profession. (Stakeholder)

I think there’s a real benefit in using a Deaf translator. A hearing interpreter has accessed English all their life so there is an English influence to their signing and sometimes intrusion from the English. A Deaf person looks at what the English means and attempts to use it visually into BSL. (Deaf community member)

## Discussion

This study aimed to explore how to most effectively deliver public health messages to sign language users and discussed the potential methods of delivering public health messages beyond direct translation. These issues were explored with members of deaf communities and also key stakeholders in Deaf communities, involved in signed languages or healthcare using small group discussions and interviews.

Findings focused on three main areas: current levels of awareness of public health messages, barriers to accessing public health messages and then views around how improvements could be facilitated.

The study found levels of awareness of health-related information to vary among members of deaf communities, but generally to be lower than hearing counterparts. Particular gaps in knowledge were noted to exist in relation to more abstract health-related concepts, mental health awareness and awareness around preventative health measures. This was found to have an impact on health behaviours and health outcomes, with anecdotes being shared highlighting subsequent harm.

A number of factors were identified as contributing to barriers in accessing and acquiring public health messages. Early life experiences were noted to contribute significantly, with many deaf children being unable to access language used in the home, contributing to difficulties with language acquisition and limited access to early fundamental health messaging. This was found to have a subsequent impact on an individual’s ability to generally grasp abstract concepts, which often limits ability to engage fully with public health messages. This agrees with previous studies which have found bilingualism acts as a protective factor to minimise the health risks faced by deaf individuals.^35 36^ Similarly, barriers pertaining to the education system were also noted to pose difficulties in terms of early acquisition of general educational messages, which form the basis on which later, more advanced, public health messaging build on. These early life experiences were noted to be compounded by ongoing lack of incidental acquisition of information, resulting in limitations to what has been termed a ‘fund of information’. It was noted that those who have limited opportunity to develop an early language around health and framework of knowledge, in which to filter and hang new information on, face more challenges in accessing and acquiring new health-related information throughout life. This can be compounded by the spread of misinformation among a relatively small community. This is made more difficult by the lack of accessible resources and difficulties in accessing health services in general.^37 38^ The COVID-19 pandemic was found to go some way to improve access for deaf communities, with the introduction of interpreters to press briefings and the commencement of a video relay service for the health service.

Discussions surrounding improving access to public health messages often focus on accessibility of the message. Participants highlighted that this is necessary but as a minimum, and accessibility alone does not necessarily equate to understanding. While ensuring English information is translated into signed languages may appear a suitable solution and is certainly necessary as a minimum, it is not adequate. Direct translation was found to be vastly unable to account for the potential existing gaps in a sign language users’ schema surrounding public health messaging, given the issues previously discussed. The study found that an attempt to improve public health awareness among deaf communities would need to be more wide-reaching and focus on impactful legislative change such as: accessibility of sign language provision for families of deaf children to support early language acquisition and improvements in education for deaf children, alongside improved accessibility. The study found that, in order to achieve meaningful wide-reaching change as opposed to ‘tokenistic accessibility’, deaf people would need to be at the forefront of this to create culturally appropriate materials and maximise impact. In order to reach all members of deaf communities, a variety of strategies and approaches should be used, rather than focus on one approach, assuming the entire community as one homogeneous group.

A strength of the study was inclusion of a broad range of members of deaf communities. A number of other stakeholders were also included. Best practice was followed in terms of provision of information about the study and gaining of informed consent (with all materials available in signed languages as well as English); inclusion of research team members with appropriate research experience but also signed languages interpreting qualifications is also a strength. Initial data collection for the study occurred just prior to the COVID-19 pandemic; the pandemic led to a temporary suspension of data collection, but that delay meant that we were able to explore how the pandemic and the need to communicate information to society broadly did lead to some shifts in information provision that benefitted deaf communities. A mixture of focus groups/small discussion groups and one-to-one interviews was chosen for efficiency and to reduce logistical challenges, but the mix of such methods may have reduced the ability to use the strength of either method (eg, within a focus group to understand social dynamics). However, each method was considered appropriate for this project as each facilitated the sharing of ideas and attitudes. The groups enabled exploration of ideas, generation of potential ideas and identification of practicalities, including facilitators and barriers, which could help generate tailored and relevant approaches in the area of the delivery of public health messages to sign language users. Much of the previous research conducted into public health message communication preferences in this community is now relatively old^22 23^ and the use of technology has advanced and also facilitated different communication methods. In addition, the field of complex intervention, including public health intervention, development has also advanced and it is now considered essential that development of such interventions requires the involvement of the target population,^33^ which has been recognised within the specific deaf community and sign language-focused literature.^11 21^ While more challenging from a communication perspective, this has to be undertaken to maximise the chances that the intervention will ultimately improve knowledge and change behaviour.

A potential weakness is also the broad range of stakeholders included and whether the views expressed differed according to the particular stakeholder group and whether they were sign language users/members of deaf communities or not. It is also less than ideal practice to have non-deaf (hearing) people speak for deaf people,^39^ yet the inclusion of those working with deaf communities in areas related to public health occurred to try to capture their input on the provision of messaging and has to be interpreted cautiously. All fieldwork was conducted in Northern Ireland, where there is the added complexity of users of signed languages employing either BSL or ISL as their first language. Given resource constraints, stakeholders were included from the predominant language used, that is, BSL, but it must be acknowledged that views may differ amongst these two different communities. The researchers who conducted the qualitative interviews were both qualified BSL interpreters, which meant that the individual session took place in only one language. However, that did therefore mean that the researchers would have known at least some of the participants—this could not have been avoided given the size of the deaf community (BSL) in Northern Ireland, but has to be acknowledged as a limitation. It is also possible that, given their embeddedness within the community, the researchers conducting the interviews and analysis of transcripts may have had certain biases regarding the topic and an enhanced, personally motivated, degree of interest in the research topic. More females were interviewed than males, and therefore, attitudes towards public health messaging will be reflected in the findings reported based on this population and may have been different if views varied between males and females. We must also consider that interviews, which took place in BSL, were translated into English prior to analysis and so the impact of the translation process must be considered. A study which takes place in its entirety in signed languages would have been logistically challenging in our setting (where the members of the research team who had more qualitative research experience, but were not BSL users) but likely beneficial and there has been discussion^40 41^ and more recent guidance emerging regarding Deaf qualitative health research.^42^ These papers consider the unique linguistic and sociopolitical considerations, and taking on board these considerations would likely reduce the chance of paternalistic and audism-riddled perspectives infiltrating findings.^40^,^42^ The decision to put all data into written English has, therefore, to be considered a weakness in the approach taken. While patient and public involvement (PPI) was undertaken in terms of development of the research question and pilot-testing of the topic guide, formal following and reporting against PPI guidelines was not conducted.^43^ PPI will be used to guide dissemination of the current manuscript in culturally appropriate ways once published. Finally, while the concepts of saturation and information power were considered in terms of the final sample size by the research team, formal assessment of these concepts did not occur, which must also be considered a weakness.

The project was designed, as is presented here, to be qualitative work around the format of public health messaging which would be used as a basis for development of new, culturally appropriate material; however, early scoping with deaf communities suggested that any information provision would be better than the status quo, which was perceived as being very limited. For that reason, we created a repository for already created material (Health Sign—Resources for Sign Language users (healthsigns.co.uk); major charity and health websites were scoped for health information available in BSL or ISL and any available videos were brought together and categorised on a newly created website. There is a need, however, to use this website as only a basis and build on it with culturally appropriate and newly developed resources. Those resources must then be kept up to date and shared widely with deaf communities. This study suggests that simple translation of public health messages will not be enough to effect increased knowledge and behaviour change in Deaf communities; messages have to be culturally adapted and the method of delivery codeveloped with members of deaf communities. The development of technology has helped with the communication challenges the community faces, but the community itself is heterogeneous, with the use of technology depending to some extent, for example, on age. Those involved in public health need to consider these complexities and account for them when developing future interventions. A next step would be the development of an intervention platform with Deaf communities and the effectiveness of this tested with these communities. More prevailing systemic issues must also be tackled to maximise impact and achieve meaningful change. There is a need for an improved education system for deaf children, greater opportunities for parents of deaf children to learn signed languages and increased accessibility for deaf communities. This will involve increasing the pool of sign language interpreters, deaf interpreters and sign language tutors. Alongside improvements in public health messaging, there must also be improvements in communication and interaction between the health service and members of deaf communities. Regular deaf awareness and basic sign language training should be encouraged.^44 45^ Vitally, those in public health should be willing to coproduce with deaf community members and consider them as experts.^46^

## Data Availability

Data are available on reasonable request from the corresponding author.
