# Supplementary material for: Delivering public health advice to sign language users: a qualitative study with key stakeholders
Source: BMJ Open. 2025 Sep 3;15(9):e098040. doi: 10.1136/bmjopen-2024-098040 (PMC12410642; doi:10.1136/bmjopen-2024-098040)
Supplement: Supplementary file 1 [file bmjopen-2024-098040.reviewer_comments.pdf]

PEER REVIEW HISTORY

BMJ Open publishes all reviews undertaken for accepted manuscripts. Reviewers are asked to complete a checklist review form and are provided with free text boxes to elaborate on their assessment. These free text comments are reproduced below.

ARTICLE DETAILS

Title (Provisional)

Delivering public health advice to sign language users: a qualitative study with key stakeholders

Authors

Rowland, Ruth; Bailey, James; Thomson, Clare; Woodside, Jayne

---

VERSION 1 - REVIEW

**Reviewer**	**1**
**Name**	**James, Tyler G**
**Affiliation**	**University of Michigan, Department of Family Medicine**
**Date**	**06-Jan-2025**
**COI**	**None**

Manuscript ID: bmjopen-2024-098040
Manuscript title: Delivering public health advice to sign language users: A qualitative study with key stakeholders

Reviewer positionality: When reviewing qualitative research, particularly with deaf communities, I find it useful to provide a positionality statement which can help editors and authors interpret my reviewer comments. I am a disabled health services research, social epidemiologist, and research methodologist in the United States. In terms of my relation to the deaf community: (1) I am not deaf, (2) both of my parents are deaf and use spoken English to communicate, (3) I am fluent in American Sign Language, and (4) I have over a decade of professional experience working in sign language interpreting and patient advocacy with Deaf and DeafBlind individuals.

MAJOR CONCERNS/COMMENTS:

- Throughout: Framing of root causes – The manuscript could do a better job at framing the root causes of language deprivation and audism as the causal mechanisms of poorer health literacy and the presence of health inequities among deaf people. The authors do this in some places, but it could be more explicit and better framed.
    - Introduction (p. 4 of 23, lines 41-60): “Communication barriers are likely to be a principal cause, but cultural and other factors may also contribute.”
    - “Additionally, many Deaf individuals grow up without the ability to overhear spoken conversations, environmental sounds or other auditory cues that

contribute to everyday knowledge acquisition. This results in a reduction in incidental learning…"

- Introduction (p. 5 of 23, lines 7-14): "…no study has engaged directly with the Deaf community to determine whether such an approach is in fact the most effective in terms of knowledge and behavior change, or whether cultural adaptation of the message would enhance their effectiveness." – There is a wealth of literature and community-based evidence reporting preferences for receiving information. It may not fall into the realm of quantitative 'effectiveness,' but the authors should do a more thorough literature review. I recommend looking at work by Kushalnagar as a starting place.
- Methods: Major points of methods reporting are missing. The authors should consult the COREQ criteria and recommended criteria for rigor and transparency in reporting qualitative research. A few areas of specific attention:
    - There are a number of qualitative approaches and study designs used in the health sciences. What was the authors' study design?
    - What was the philosophical orientation of the study (e.g., epistemological and ontological assumptions)? This is <u>critical</u> considering the mix of participants – some of whom are not signed language users. How are the authors ensuring paternalistic and audism-riddled perspectives are not infiltrating the findings?
    - In terms of sample size sufficiency, the authors need to indicate what they mean by "data saturation" and "information power." How were these determined and assessed? Why were they chosen? (e.g., saturation has a strong history in grounded theory studies.)
    - What was the prior relation between researchers and participants?
    - What is the researchers' positionality? This is particularly important given the power dynamics between interpreters and deaf people.
    - How many researchers coded the transcripts? Was a codebook never created and revised?
    - The mixing of focus group and interview data needs to be justified and further considered. In focus group research, typically the focus group is the unit of analysis – since focus groups are used to understand social dynamics.
    - Were interviews / focus groups conducted directly in BSL/ISL, or spoken and interpreted?
- Results: The authors should consult qualitative methodological guidance on the development of themes in thematic analysis, e.g., Saldaña's 'touch test.' Themes should be descriptive and conclusive sentences, and provide something new to the field – while demonstrating the effort and work put into the study. The current themes are undescriptive. Further, the results are reported such that the reader is responsible for additional interpretation of a long listing of quotations.
- Results: When reading through the results, I wonder if the authors are trying to report too much. For example, is the section on early life experiences really relevant to providing pragmatic recommendations on how to deliver public health communication?
- Discussion: Is the inclusion of broad range of stakeholders a strength of the study? How does it violate Deaf epistemologies by having non-deaf (hearing) people speak for deaf people?
- Discussion: The section on focus groups being "valid and efficient" isn't a specific strength. Validity is context dependent, and efficiency is traded off for depth.
- Discussion: In the manuscript, the authors state "writing up qualitative research in a language which interviews did not take place in poses inherent challenges" – I expected more discussion on this point. In addition, I think that authors should disclose if they coded textual (English translated) data, or video data. (See Anderson et al. for more information.)

OTHER CONCERNS/COMMENTS:

- Throughout: "Deaf community" or "Deaf communities" – The authors may want to review the work of deaf researchers discussing heterogeneity among deaf communities, and avoiding the term "Deaf community" as it insinuates a monolith.
- Throughout: Capitalization of "Sign Language" versus using "signed language" – In my 10 years of conducting deaf health research, I have never seen "Sign Language" as a way to refer to multiple signed languages. Typically, what is done in sociolinguistics – and adopted by the health sciences – is talking about "signed languages."
- Methods: "balance across sex was not achieved" – why is this important / why was it a target?

**Reviewer**	**2**
**Name**	**Young, Alys**
**Affiliation**	**University of Manchester, Oxford Road, M13 9PL, Manchester, UK, Health Sciences**
**Date**	**20-Jan-2025**
**COI**	**None**

It is always good to see a research study in this field of inquiry and it is very good to see, I believe for the first time, something like this specific to the Northern Ireland context. This is however one of a great many studies being carried out world wide that are concerned with differing aspects of inequalities experienced by Deaf signers in relation to health access, treatment and outcomes that encompass also concerns about health literacy and public health messaging. It would therefore have been more appropriate to see some of this literature referenced in the introductory section in addition to outlining some of the well known difficulties faced concerning language, service provision, access and cultural-linguistically inappropriate provisions.

What is potentially novel about this paper is the focus on how to most effectively deliver public health messages to Sign Language users and the potential methods of delivering public health messages beyond direct translation. That for me is the nub of this paper yet there is no review of the various studies and publications out there have expressly tackled this issue (or tried to). The paper drifts instead into more general concerns about health access, language barriers and inflexible structures of provision for deaf people. It would have been better to keep the paper focussed clearly on the specific concern stated as the aim to explore. The addition of an aim that arose because of the circumstances of the pandemic was interesting but again my suggestion is either do that fully or subsume it in the first aim. This is because there is no mention of the growing literature on health messaging and Deaf people linked with the pandemic and more widely of the 'gains' of online approaches to communication being mainstreamed through the pandemic that incidentally have both benefited and 'disabled' deaf people with regard to health. Addressing issues like remote interpreting (which is about access really not about public health messaging) is a distraction

especially as the authors do not review or cite any of large literature on this in relation to Deaf people, especially from the US and Europe.

If the paper were streamlined both in its introductory literature and its findings this would be a more novel contribution. The quotations and the length of the findings could be reduced and more targeted. When topics about why simple translation is not enough and what would be a deaf led targeted campaign are discussed, this is much stronger and potentially novel. A lot of what is reported, especially in the barriers to accessing services sections, seems secondary (although important) to the main focus of the work.

Placing the discussion and the recommendations in a wider global context of the vast amount of work going on about inclusion of d/Deaf people, improving d/Deaf health literacy and experimental studies in interventions in health in signed languages (of which there are several going on both in the UK and world wide) would have placed the findings in a more appropriate context.

The methods section requires some considerable revision as it does not meet the quality standards in qualitative reporting on either of the EQUATOR recommended checklists. Specifically the lack of information about methodological basis of the inquiry is of concern. Calling it qualitative is not enough. The approach to data analysis is only scantily referenced to any particular approach with few details on the management of the data and the progression of the inductive analysis. If Braun and Clarke were used, were all steps in their process followed or modified? There is almost no reflexive analysis anywhere and the identities, language biographies, health status, professional background etc of the researchers is very opaque yet all took part in the inductive analysis. The researchers treat the translation of all data into one language as self evident. It is not. This is bilingual bicultural research project and a deliberate decision was made to keep in one language and transcribe rather than use the direct source languages – why? What were the effects? There is a literature on this and specifically in relation to signed languages too. See Temple, B., Young, A.M. (2004) Qualitative Research and Translation Dilemmas. Qualitative Research, 4 (2), 161 – 178. doi: 10.1177/1468794104044430 and also Young and Temple (2014) Approaches to Social Research: the case of Deaf studies (there is a whole chapter on this topic).

Authors may want to look at a recent systematic review that may be helpful be it focused on health outcomes rather than public health: Rogers, K., Rowlandson, A., Harkenss, J., Shields, G., Young. A. (2024). Health outcomes in Deaf signing populations: a systematic review. Plos One. https://doi.org/10.1371/journal.pone.0298479

The following may also be helpful although tangential perhaps the reference lists contain literature it may be wroth consulting:

Ferguson-Coleman, E., Young, A. (2023). “'What have YOU done in the past few years?’: Deaf BSL users' experiences caring for people with dementia during COVID-19”. Quality in Ageing and Older Adults. https://doi.org/10.1108/QAOA-07-2023-0048

Young, A., Ferguson-Coleman, E., Keady, J. (2019). How might the cultural significance of storytelling in Deaf communities influence the development of a life story work intervention for Deaf people with dementia?: A conceptual thematic review. Ageing and Society 40 (2), 262 – 281. https://doi.org/10.1017/S0144686X18000946

Young, A., Ferguson-Coleman, E., Keady, J. (2018). Authentic Public and Patient Involvement with Deaf sign language users: it is not just about language access. Dementia, 17 (8), 1001 – 1010. https://doi.org/10.1177/1471301218789567

Ferguson-Coleman, E., Keady, J., Young, A. (2014). Dementia and the Deaf community: knowledge and service access. Aging and Mental Health, 18 (6), 674-682. doi: 10.1080/13607863.2014.880405
https://www.tandfonline.com/doi/abs/10.1080/13607863.2014.880405

VERSION 1 - AUTHOR RESPONSE

Reviewer 1

Manuscript ID: bmjopen-2024-098040

Manuscript title: Delivering public health advice to sign language users: A qualitative study with key stakeholders

Reviewer positionality: When reviewing qualitative research, particularly with deaf communities, I find it useful to provide a positionality statement which can help editors and authors interpret my reviewer comments. I am a disabled health services research, social epidemiologist, and research methodologist in the United States. In terms of my relation to the deaf community: (1) I am not deaf, (2) both of my parents are deaf and use spoken English to communicate, (3) I am fluent in American Sign Language, and (4) I have over a decade of professional experience working in sign language interpreting and patient advocacy with Deaf and DeafBlind individuals.

Thanks for this positionality statement and the very helpful review of the manuscript – we have responded, making changes where possible and outline these below.

MAJOR CONCERNS/COMMENTS:

• Throughout: Framing of root causes – The manuscript could do a better job at framing the root causes of language deprivation and audism as the causal mechanisms of poorer health literacy

and the presence of health inequities among deaf people. The authors do this in some places, but it could be more explicit and better framed.

- Introduction (p. 4 of 23, lines 41-60): “Communication barriers are likely to be a principal cause, but cultural and other factors may also contribute.”
- “Additionally, many Deaf individuals grow up without the ability to overhear spoken conversations, environmental sounds or other auditory cues that contribute to everyday knowledge acquisition. This results in a reduction in incidental learning…”

We agree that this was not presented as clearly as it could have been and have therefore revised the introduction to frame the root causes more clearly in the paper, as requested by the reviewer. We have also revised the discussion accordingly.

• Introduction (p. 5 of 23, lines 7-14): “…no study has engaged directly with the Deaf community to determine whether such an approach is in fact the most effective in terms of knowledge and behavior change, or whether cultural adaptation of the message would enhance their effectiveness.” – There is a wealth of literature and community-based evidence reporting preferences for receiving information. It may not fall into the realm of quantitative ‘effectiveness,’ but the authors should do a more thorough literature review. I recommend looking at work by Kushalnagar as a starting place.

This has been more carefully stated and the relevant literature included.

• Methods: Major points of methods reporting are missing. The authors should consult the COREQ criteria and recommended criteria for rigor and transparency in reporting qualitative research.

The COREQ criteria have now been checked and a checklist completed, as has the SRQR checklist, as requested by the journal.

A few areas of specific attention:

o There are a number of qualitative approaches and study designs used in the health sciences. What was the authors’ study design?

The study design was phenomenological in approach, which is now included in the text, usually exploring human experiences related to a particular phenomenon – which in this case is access to public health messaging in signed language users.

o What was the philosophical orientation of the study (e.g., epistemological and ontological assumptions)? This is critical considering the mix of participants – some of whom are not signed language users. How are the authors ensuring paternalistic and audism-riddled perspectives are not infiltrating the findings?

Given the ambition of developing practical understandings of concrete, real-world issues around public health messaging in Deaf communities, the philosophical orientation can be considered pragmatic and this is now included, but the potential impact of this is acknowledged in the discussion.

o In terms of sample size sufficiency, the authors need to indicate what they mean by "data saturation" and "information power." How were these determined and assessed? Why were they chosen? (e.g., saturation has a strong history in grounded theory studies.)

Information on these concepts has been added, but these were not formally assessed, and this has been noted as a weakness.

o What was the prior relation between researchers and participants?

As already explained in the manuscript, initial interviews were conducted by one qualitative researcher and a second set of interviews conducted post-COVID-19 pandemic were conducted by a second qualitative researcher. Each of these researchers was a qualified BSL interpreter, which meant that the individual session took place in only one language. However that did therefore mean that the researchers will have known at least some of the participants – that could not have been avoided give the size of the Deaf community in Norther Ireland, but we have acknowledged this as a limitation within the discussion.

o What is the researchers' positionality? This is particularly important given the power dynamics between interpreters and deaf people.

The positionality of the researchers who were in direct contact with the study participants is now given within the manuscript.

o How many researchers coded the transcripts? Was a codebook never created and revised?

More detailed methodology has now been included re: transcript coding.

o The mixing of focus group and interview data needs to be justified and further considered. In focus group research, typically the focus group is the unit of analysis – since focus groups are used to understand social dynamics.

A mix of focus groups and interviews was conducted, purely for efficiency and logistical reasons (which also fits with the pragmatism approach as already outlined). We acknowledge that that may not have been optimal and now highlight this within the discussion.

o Were interviews / focus groups conducted directly in BSL/ISL, or spoken and interpreted?

Interviews and focus groups were conducted directly in BSL, where that was the preference of the participant and this is included in the methods.

• Results: The authors should consult qualitative methodological guidance on the development of themes in thematic analysis, e.g., Saldaña's 'touch test.' Themes should be descriptive and conclusive sentences, and provide something new to the field – while demonstrating the effort and work put into the study. The current themes are undescriptive. Further, the results are reported such that the reader is responsible for additional interpretation of a long listing of quotations.

We have revisited the themes and how they are worded and developed them. We have also revised the reporting, including some of the more generic material into Supplementation information so we hope that has helped with interpretability. We are very happy to make further edits should the reviewer request it.

• Results: When reading through the results, I wonder if the authors are trying to report too much. For example, is the section on early life experiences really relevant to providing pragmatic recommendations on how to deliver public health communication?

We thank the reviewer for this helpful comment and agree that we tried to include the full content of the data collected. We have now, based on both review comments, revised the aim, and reduced the content that is less relevant to the main aim of the paper.

• Discussion: Is the inclusion of broad range of stakeholders a strength of the study? How does it violate Deaf epistemologies by having non-deaf (hearing) people speak for deaf people?

We have revised the text here to better reflect the intended meaning i.e. that we attempted to capture the heterogeneity of Deaf communities and we also acknowledge the challenges and likely implications of including non-deaf (hearing) people in the sample and describe the rationale for doing this.

• Discussion: The section on focus groups being “valid and efficient” isn’t a specific strength. Validity is context dependent, and efficiency is traded off for depth.

We acknowledge that this should not have been portrayed as a strength and highlight the potential weaknesses of our approach.

• Discussion: In the manuscript, the authors state “writing up qualitative research in a language which interviews did not take place in poses inherent challenges” – I expected more discussion on this point. In addition, I think that authors should disclose if they coded textual (English translated) data, or video data. (See Anderson et al. for more information.)

This detail has now been included and more discussion and relevant references also included.

OTHER CONCERNS/COMMENTS:

• Throughout: “Deaf community” or “Deaf communities” – The authors may want to review the work of deaf researchers discussing heterogeneity among deaf communities, and avoiding the term “Deaf community” as it insinuates a monolith.

We acknowledge that the term Deaf communities is more appropriate and have now included this term instead of the singular Deaf community, unless this was the terminology used within quotations from the data collected as part of the study (where singular Deaf community was frequently used, likely due to the small geographic region focused on within the data collection).

• Throughout: Capitalization of “Sign Language” versus using “signed language” – In my 10 years of conducting deaf health research, I have never seen “Sign Language” as a way to refer to multiple signed languages. Typically, what is done in sociolinguistics – and adopted by the health sciences – is talking about “signed languages.”

Throughout the manuscript the term Sign Language has been replaced, as appropriate, with "signed language". Where this occurs within quotations from focus groups or interviews, this has been left as is. Much of the focus here was on BSL, but that is also now discussed within the manuscript.

• Methods: "balance across sex was not achieved" – why is this important / why was it a target?

This has been removed from both the methods and the Article Summary section.

Reviewer 2

Reviewer: 2
Dr. Alys Young, University of Manchester, Oxford Road, M13 9PL, Manchester, UK
Comments to the Author:
It is always good to see a research study in this field of inquiry and it is very good to see, I believe for the first time, something like this specific to the Northern Ireland context. This is however one of a great many studies being carried out world wide that are concerned with differing aspects of inequalities experienced by Deaf signers in relation to health access, treatment and outcomes that encompass also concerns about health literacy and public health messaging. It would therefore have been more appropriate to see some of this literature referenced in the introductory section in addition to outlining some of the well known difficulties faced concerning language, service provision, access and cultural-linguistically inappropriate provisions.

We thank the reviewer for their helpful observations and have tried to include more recent, relevant material throughout the manuscript and thank them for pointing out specific references below.

What is potentially novel about this paper is the focus on how to most effectively deliver public health messages to Sign Language users and the potential methods of delivering public health messages beyond direct translation. That for me is the nub of this paper yet there is no review of the various studies and publications out there have expressly tackled this issue (or tried to). The paper drifts instead into more general concerns about health access, language barriers and inflexible structures of provision for deaf people. It would have been better to keep the paper focussed clearly on the specific concern stated as the aim to explore. The addition of an aim that arose because of the circumstances of the pandemic was interesting but again my suggestion is either do that fully or subsume it in the first aim. This is because there is no mention of the growing literature on health messaging and Deaf people linked with the pandemic and more widely of the 'gains' of online approaches to communication being mainstreamed through the pandemic that incidentally have both benefited and 'disabled' deaf people with regard to health. Addressing issues like remote interpreting (which is about access really not about public health messaging) is a distraction especially as the authors do not review or cite any of large literature on this in relation to Deaf people, especially from the US and Europe.

We thank the reviewer for their helpful suggestions and agree. The issue of including too much information and diluting the main aim has also been suggested by the other reviewer. We have

therefore revised our aim and data presentation accordingly and reduce any additional information – including some of this within supplementary information. We hope the reviewer considers that this has helped the data presentation and made the main focus of the paper clearer.

If the paper were streamlined both in its introductory literature and its findings this would be a more novel contribution. The quotations and the length of the findings could be reduced and more targeted. When topics about why simple translation is not enough and what would be a deaf led targeted campaign are discussed, this is much stronger and potentially novel. A lot of what is reported, especially in the barriers to accessing services sections, seems secondary (although important) to the main focus of the work.

As above, we agree and have tried to revise and reduce our results, as well as refine the introduction, to reflect this.

Placing the discussion and the recommendations in a wider global context of the vast amount of work going on about inclusion of d/Deaf people, improving d/Deaf health literacy and experimental studies in interventions in health in signed languages (of which there are several going on both in the UK and world wide) would have placed the findings in a more appropriate context.

We have tried to bring in this literature as suggested.

The methods section requires some considerable revision as it does not meet the quality standards in qualitative reporting on either of the EQUATOR recommended checklists. Specifically the lack of information about methodological basis of the inquiry is of concern. Calling it qualitative is not enough. The approach to data analysis is only scantily referenced to any particular approach with few details on the management of the data and the progression of the inductive analysis. If Braun and Clarke were used, were all steps in their process followed or modified? There is almost no reflexive analysis anywhere and the identities, language biographies, health status, professional background etc of the researchers is very opaque yet all took part in the inductive analysis.

We have now included the COREQ and SRQR checklists as suggested by both reviewers and the journal and this, alongside responses to both reviewers, means we believe that all of this information is now included.

The researchers treat the translation of all data into one language as self evident. It is not. This is bilingual bicultural research project and a deliberate decision was made to keep in one language and transcribe rather than use the direct source languages – why? What were the effects? There is a literature on this and specifically in relation to signed languages too. See Temple, B., Young, A.M. (2004) Qualitative Research and Translation Dilemmas. Qualitative Research, 4 (2), 161 – 178. doi: 10.1177/1468794104044430 and also Young and Temple (2014) Approaches to Social Research: the case of Deaf studies (there is a whole chapter on this topic).

This detail has now been included and more discussion and relevant references also included.

Authors may want to look at a recent systematic review that may be helpful be it focused on health outcomes rather than public health: Rogers, K., Rowlandson, A., Harkenss, J., Shields, G., Young. A. (2024). Health outcomes in Deaf signing populations: a systematic review. Plos One. https://doi.org/10.1371/journal.pone.0298479

We thank the reviewer as we were not aware of this manuscript which has been very useful.

The following may also be helpful although tangential perhaps the reference lists contain literature it may be wroth consulting:
Ferguson-Coleman, E., Young, A. (2023). "'What have YOU done in the past few years?': Deaf BSL users' experiences caring for people with dementia during COVID-19". Quality in Ageing and Older Adults. https://doi.org/10.1108/QAOA-07-2023-0048
Young, A., Ferguson-Coleman, E., Keady, J. (2019). How might the cultural significance of storytelling in Deaf communities influence the development of a life story work intervention for Deaf people with dementia?: A conceptual thematic review. Ageing and Society 40 (2), 262 – 281. https://doi.org/10.1017/S0144686X18000946
Young, A., Ferguson-Coleman, E., Keady, J. (2018). Authentic Public and Patient Involvement with Deaf sign language users: it is not just about language access. Dementia, 17 (8), 1001 – 1010. https://doi.org/10.1177/1471301218789567
Ferguson-Coleman, E., Keady, J., Young, A. (2014). Dementia and the Deaf community: knowledge and service access. Aging and Mental Health, 18 (6), 674-682. doi: 10.1080/13607863.2014.880405 https://www.tandfonline.com/doi/abs/10.1080/13607863.2014.880405

As above, we have consulted these papers and brought in references cited as appropriate as well as conducting a thorough recent literature search, which we believe has benefitted the paper.

VERSION 2 - REVIEW

**Reviewer**	**2**
**Name**	**Young, Alys**
**Affiliation**	**University of Manchester, Oxford Road, M13 9PL, Manchester, UK, Health Sciences**
**Date**	**29-Apr-2025**
**COI**	

Abstract: Requires further revision.

The opening statement about the size of the Deaf community in the uK is inaccurate for two reasons: Deaf identity is not a product of how deaf someone is therefore to link it the number of profoundly deaf people is not correct. Second to say that many of the 800,000 are members of the Deaf community in the UK is wrong. Why not use the figures from the British Deaf Association – roughly 120,000

This sentence is ambiguous because it is not clear what in the previous sentences the 'this' refers to: "Studies of health behaviour and knowledge and health related attitudes and beliefs show this to be sub-optimal amongst Deaf and hard of hearing individuals." The previous sentence is about membership of the Deaf community so are you saying being a member is sub-optimal. I don't think so. The next sentence compounds this further as again the 'this' is unclear and sub optimal for what? "The reasons for this are not well understood".

The opening paragraph adopts the 'signed language users' convention but the second paragraph return to 'Sign Language users'.

Under design – if you have corrected this in the main body to be more specific than just 'qualitative' why is not corrected in the abstract?

Under participants you are pluralising deaf communities in Northern Ireland. Is that because you are making a distinction on sectarian and language lines? Or are you simply following the suggestion of one of the reviewers to indicate plurality. It is much more usual in the UK content to talk about the Deaf community as an entity then quality that such as the Deaf Community in Northern Ireland. I have seen deaf communities in NI used but usually only to make the point that the sample is drawn from BSL and ISL using groups.

" There were 16 focus groups or one-to-one interviews held…" – why not just say how many focus groups and how many individual interviews before stating the total number of participants. Does not seem to have any value in being general here. Much more usual to say Deaf community members not deaf communities' members unless you really are making a key distinction between specifically different groups within NI. I would also like to know how many of the sample are from the Deaf community and how many external stakeholders not just the language distribution. Why shy away from that?

Terminology: I can see the authors have tried to take on board the notion of plurality in sign languages and their users but it has resulted in rather confused and clumsy usage and terminology. I suggest a review again of the authors' terminology. This is my rule of thumb.

'sign language' is the noun that refers to a language. 'Signed language' is adjectival like hearing people use 'spoken' language with spoken/signed acting as the qualifier to the noun language. So for example you have multiple sign languages and sign language users (like French language users). You can have 'information produced in signed languages' or 'users of signed language' but not really signed language users. When I write about a country with more than one sign language within it I would also use for example 'sign language(s)'.

Usually Deaf Community is singular with the qualifier for multiple communities happening after it such as ‘the two main Deaf communities in Canada’ or single Deaf community but with acknowledgement of intersectional features e.g. ‘Asian Deaf community in the UK’ or ‘Deaf women in the community’ But one would say ‘Deaf communities worldwide’ for example when it is really important not to assume there is only one international community. But equally you can write about trans-national Deaf communities when emphasising features that are in common with being Deaf rather than national identity or which particular sign language is being used.

Strengths and limitations

These are improved with one exception: “• Interviews which took place in British Sign Language (BSL) required translation to English for subsequent transcription and write up therefore . Writing up qualitative research in a language in which not all interviews took did not take place, which in poses inherent challenges.” I challenge the notion that translation was ‘required’ for transcription. It is a choice the researchers made, not a requirement. There are plenty of examples of qualitative data being kept in the source sign language for purposes of analysis. I suggest this is rewritten to firmly acknowledge that the chose to translate (not just transcribe) BSL data results in engaging with translated text rather than source language that creates challenges of not just linguistic understanding but also cultural representation.

My point also applies to the main text concerning the methods section where the notion of translation for transcription purposes without any7 account of the potential losses involved in this is given. In my previous reviewer comments I pointed the authors to several discussions of this point and it remains a surprising choice still given the fluency of the researchers in BSL. Therefore I am requesting again some justification for the choice to put all data into written English (and for transcription purposes is not a methodological justification) and to at least reference that the choice to do this can be contentious.

PPIE – it is good to see some explanation of this but perhaps consider referencing the description of what you have done within the GRIPP2 reporting guidelines. This is not a requirement just a suggestion to strengthen that part of the paper.

Thematic analysis: It is good to see greater detail addressing the coding process. I wonder why NVIVO or similar was not used in preference to Excel given the details about how the work was done. Seems a bit odd. Maybe worth a comment from the authors in the text why NVIVO was not used given its advantages from a validity perspective. However there is still a gap between description of the coding process and the construction of the themes that is not explained. If the Braun and Clarke model was used perhaps that would be helpful in filling that gap. Simply saying themes were constructed is usually not enough specifically as the authors are saying this is a phenomenological approach.

Findings: This is much better, well targeted and more directly addressing the public health aspects. However, I think it would be helpful to know whether the directly quoted texts

came from members of the Deaf community or from stakeholders. These are very different perspectives within the group of participants potentially and yet they are put together as one group.

The discussion is more scholarly and measured than previously.

Supplementary material – my personal view is that the small amount of additional data in the supplementary materials is not necessary.

VERSION 2 - AUTHOR RESPONSE

Reviewer: 2
Dr. Alys Young, University of Manchester, Oxford Road, M13 9PL, Manchester, UK
Comments to the Author:
Abstract: Requires further revision.
The opening statement about the size of the Deaf community in the uK is inaccurate for two reasons: Deaf identity is not a product of how deaf someone is therefore to link it the number of profoundly deaf people is not correct. Second to say that many of the 800,000 are members of the Deaf community in the UK is wrong. Why not use the figures from the British Deaf Association – roughly 120,000

This edit has been made – many thanks for the suggestion.

This sentence is ambiguous because it is not clear what in the previous sentences the 'this' refers to: "Studies of health behaviour and knowledge and health related attitudes and beliefs show this to be sub-optimal amongst Deaf and hard of hearing individuals." The previous sentence is about membership of the Deaf community so are you saying being a member is sub-optimal. I don't think so. The next sentence compounds this further as again the 'this' is unclear and sub optimal for what? "The reasons for this are not well understood".
The opening paragraph adopts the 'signed language users' convention but the second paragraph return to 'Sign Language users'.

These sentences have been edited – many thanks for the careful scrutiny of language used.

Under design – if you have corrected this in the main body to be more specific than just 'qualitative' why is not corrected in the abstract?

Edit completed.

Under participants you are pluralising deaf communities in Northern Ireland. Is that because you are making a distinction on sectarian and language lines? Or are you simply following the suggestion of one of the reviewers to indicate plurality. It is much more usual in the UK content to talk about the Deaf community as an entity then quality that such as the Deaf Community in Northern Ireland. I have seen deaf communities in NI used but usually only to make the point that the sample is drawn from BSL and ISL using groups.

This edit was in response to a previous reviewer and we have made further edits, which we agree are more clear, to resolve this.

" There were 16 focus groups or one-to-one interviews held..." – why not just say how many focus groups and how many individual interviews before stating the total number of participants. Does not seem to have any value in being general here. Much more usual to say Deaf community members not deaf communities' members unless you really are making a key distinction between specifically different groups within NI. I would also like to know how many of the sample are from the Deaf community and how many external stakeholders not just the language distribution. Why shy away from that?

This detail has been added as requested.

Terminology: I can see the authors have tried to take on board the notion of plurality in sign languages and their users but it has resulted in rather confused and clumsy usage and terminology. I suggest a review again of the authors' terminology. This is my rule of thumb. 'sign language' is the noun that refers to a language. 'Signed language' is adjectival like hearing people use 'spoken' language with spoken/signed acting as the qualifier to the noun language. So for example you have multiple sign languages and sign language users (like French language users). You can have 'information produced in signed languages' or 'users of signed language' but not really signed language users. When I write about a country with more than one sign language within it I would also use for example 'sign language(s)'. Usually Deaf Community is singular with the qualifier for multiple communities happening after it such as 'the two main Deaf communities in Canada' or single Deaf community but with acknowledgement of intersectional features e.g. 'Asian Deaf community in the UK' or 'Deaf women in the community' But one would say 'Deaf communities worldwide' for example when it is really important not to assume there is only one international community. But equally you can write about trans-national Deaf communities when emphasising features that are in common with being Deaf rather than national identity or which particular sign language is being used.

Many thanks for this helpful comment and examples – we have worked through the manuscript and believe we have edited for clarity throughout – you are quite right the previous edits were in response to another reviewer comments so do appreciate you making further suggestions. We are happy to make any further specific changes as required.

Strengths and limitations
These are improved with one exception: "• Interviews which took place in British Sign Language (BSL) required translation to English for subsequent transcription and write up therefore . Writing up qualitative research in a language in which not all interviews took did not take place, which in poses inherent challenges." I challenge the notion that translation was 'required' for transcription. It is a choice the researchers made, not a requirement. There are plenty of examples of qualitative data being kept in the source sign language for purposes of analysis. I suggest this is rewritten to firmly acknowledge that the chose to translate (not just transcribe) BSL data results in engaging with translated text rather than source language that creates challenges of not just linguistic understanding but also cultural representation.

This has been rewritten as suggested.

My point also applies to the main text concerning the methods section where the notion of translation for transcription purposes without any account of the potential losses involved in this is given. In my previous reviewer comments I pointed the authors to several discussions of this point and it remains a surprising choice still given the fluency of the researchers in BSL. Therefore I am requesting again some justification for the choice to put all data into written English (and for transcription purposes is not a methodological justification) and to at least reference that the choice to do this can be contentious.

This has been completed.

PPIE – it is good to see some explanation of this but perhaps consider referencing the description of what you have done within the GRIPP2 reporting guidelines. This is not a requirement just a suggestion to strengthen that part of the paper.

The PPIE section and other parts of the manuscript include the main elements of the GRIPP2 short form, and the guidance referred to, including the limitations of the PPIE undertaken and future plans.

Thematic analysis: It is good to see greater detail addressing the coding process. I wonder why NVIVO or similar was not used in preference to Excel given the details about how the work was done. Seems a bit odd. Maybe worth a comment from the authors in the text why NVIVO was not used given its advantages from a validity perspective.

A rationale for not using NVIVO is now included.

However there is still a gap between description of the coding process and the construction of the themes that is not explained. If the Braun and Clarke model was used perhaps that would be helpful in filling that gap. Simply saying themes were constructed is usually not enough specifically as the authors are saying this is a phenomenological approach.

This section has been rewritten with direct reference to the Braun and Clarke model.

Findings: This is much better, well targeted and more directly addressing the public health aspects. However, I think it would be helpful to know whether the directly quoted texts came from members of the Deaf community or from stakeholders. These are very different perspectives within the group of participants potentially and yet they are put together as one group.

The source of the quotes is now given in the text.

The discussion is more scholarly and measured than previously.

Many thanks for this comment.

Supplementary material – my personal view is that the small amount of additional data in the supplementary materials is not necessary.
We are happy to remove the Supplementary material if the reviewer thinks it does not add value.

Reviewer: 2

If you have selected ‘Yes’ above, please provide details of any competing interests.: not applicable

Many thanks – we believe our previous responses have satisfied this reviewer.

VERSION 3 - REVIEW

Reviewer 2

Name Young, Alys

Affiliation University of Manchester, Oxford Road, M13 9PL, Manchester, UK, Health Sciences

Date 14-Aug-2025

COI

The authors have done a very good and detailed job of responding to the detailed comments of the reviewers. I hope they can see how the manuscript has substantially improved and I am confident now it meets the required standard for publication. I welcome this work as an addition to the literature in this area of study.
